# A Review of Food Frequency Questionnaires Developed  and Validated in Japan

**DOI:** 10.2188/jea.JE20081007

**Published:** 2009-01-30

**Authors:** Kenji Wakai

**Affiliations:** 1 Department of Preventive Medicine/Biostatistics and Medical Decision Making, Nagoya University Graduate School of Medicine, Nagoya, Japan

**Keywords:** food frequency questionnaire, validity, reproducibility, nutrient, food group

## Abstract

**Background:**

The food frequency questionnaire (FFQ) has been used throughout the world for epidemiological purposes. Because dietary habits vary greatly, the FFQ must be tailored for use with specific populations. The usefulness of FFQs in Japan was assessed by reviewing questionnaires developed and validated in that country.

**Methods:**

A literature search was conducted to identify articles on the development and/or validation of FFQs for Japanese populations. For each FFQ identified, validation studies were used to abstract its characteristics and information. The correlation coefficients between diet records (DRs) and FFQ estimates and those between the same FFQs completed twice were used to evaluate validity and reproducibility, respectively, of the questionnaires.

**Results:**

Twenty-one eligible FFQs were identified. They were found to be reasonably valid and reproducible. The median of correlation coefficients between DRs and FFQs ranged from 0.31 to 0.56 for target nutrients, and that between the same FFQs completed twice within a period of 9 months to 1 year ranged from 0.50 to 0.72. Relatively poor validity was found for FFQ estimates on consumption of potatoes, seaweed, sodium, niacin, and polyunsaturated fatty acids. For the purpose of analysis, FFQs were divided into long FFQs (97 or more food items) and short FFQs (<70 items); the former had slightly higher validity.

**Conclusion:**

FFQs are useful for assessing dietary intake in Japan, although careful consideration is required for the food groups and nutrients for which FFQs had low validity.

## INTRODUCTION

In epidemiological studies that attempt to elucidate the relations between diet and chronic disease, the methods used to assess the diets of participants must be valid, but also inexpensive. In addition, they should not place a heavy burden on either the participants or the research staff. Finally, it must be possible to collect information on usual or average diet over an extended period, rather than a period of just a few days.

The food frequency questionnaire (FFQ) has been widely used for epidemiological purposes because it satisfies the above conditions.^1^ In FFQs, selected food items are listed, and the intake frequencies and usual portion or serving sizes (average quantity of foods per intake) are noted. The consumption of a food item is estimated by multiplying the portion size by its intake frequency. Nutrient intakes can also be assessed by establishing a database of compositions of the food items for target nutrients. The intake of a nutrient is calculated using the following formula for each item: (reported intake frequency per day) × (portion size in grams) × (nutrient content per 100 grams)/100. The intake is then summed for all the listed food items to obtain the intake per day. Information on portion sizes is not collected in some FFQs; instead, standard sizes are used to estimate food or nutrient intake.

Because dietary habits vary greatly depending on the ethnic, social, and cultural background of participants, FFQs must be tailored to target populations. For example, the food items in FFQs should reflect the dietary habits of participants. Many FFQs for Japanese populations have been developed in the last 2 decades.^2^^–^^35^ They have been validated by comparing responses to the questionnaire with participants' actual diets, as recorded for a few to dozens of days. The author and collaborators also developed a FFQ^19^^,^^20^ and have applied it to epidemiological studies.^36^^–^^40^ In this article, FFQs that were developed and validated in Japan were examined to assess the usefulness of such questionnaires in that country.

## METHODS

A MEDLINE search was conducted using PubMed to identify articles on the development or validation of FFQs for Japanese populations from 1980 to June 2008. The query used for the searches was “food frequency AND Japan AND (validity OR reproducibility)”. In addition, the author manually searched references from relevant articles where necessary. Papers written in either English or Japanese were reviewed. For each FFQ identified, the author abstracted its characteristics, including the numbers of food items and response categories for intake frequency, questions on portion sizes, methods of questionnaire development, and major studies in which the questionnaire was used.

To determine the validity of FFQs, the correlation coefficients between diet records (DRs) and FFQ estimates were collected from the articles. Data from both nutrients and food groups were examined, together with data on the participants in the validation study, the period of the DR that was used as a reference, the number of nutrients validated, and the energy adjustment and de-attenuation of the coefficients. As for reproducibility, the correlation coefficients between identical FFQs that were completed twice, and the interval between the completion dates, were abstracted for each FFQ.

The medians were computed when the correlation coefficients for validity or reproducibility were summarized over nutrients or FFQs. When 2 or more correlation coefficients (ie, those for sex and/or population) were available for 1 FFQ, their median was used as the representative value for that FFQ.

## RESULTS

### FFQs developed and validated in Japan

The literature search identified 21 FFQs that were developed and validated in Japan (FFQ Nos. in Table 1: 1–21). They are listed in Table 1, with their selected characteristics, in the order of the number of food items included, which ranged from 9 to 169. Two questionnaires were developed specifically to estimate the dietary intake of calcium (No. 5) or calcium plus other nutrients relevant to osteoporosis (No. 6). Two FFQs validated by Yatsuya et al (No. 1) and Sauvaget et al (No. 3) were used solely to assess the consumption of individual foods or food groups (eg, fish, vegetables, fruit). Another 17 FFQs were used to estimate the intakes of comprehensive sets of nutrients.

**Table 1.** Characteristics of food frequency questionnaires developed and validated in Japan (sorted by number of food items included)*

No.	Authors of references	No. of food items	No. of response categories for intake frequency	Questions on portion sizes	Method of development	References		Main studies	Comments

						Development	Validation†		
1	Yatsuya et al.	9	4	No	Experience-based		2		
2	Nakamura et al.	21	Open-ended	Yes	Experience-based		3		Intakes of foods were queried for breakfast, lunch, and dinner, separately.
3	Sauvaget et al.	22	4	No	Experience-based		4	Life Span Study	
4	Katagiri et al.	24	6	Yes	Experience-based		5		Questionnaire completed by an interviewer
5	Sato et al.	26	NA	Yes	Experience-based	6	6	JPOS Study	FFQ to estimate dietary intake of calcium
6	Uenishi et al.	28	3–5	Yes	Experience-based	7	7		FFQ to estimate dietary intake of calcium and other nutrients relevant to osteoporosis.
7	Takatsuka et al.	31	NA	NA	Short version	8	8		
8	Ogawa et al.	40	5	No‡	Experience-based		9	Miyagi Cohort, Ohsaki Cohort	
9	Date et al.	40	5	No‡	Experience-based		10	JACC	Almost the same FFQ as that validated by Ogawa et al.
10	Tsugane et al.	44	4	No‡	Experience-based		11, 12	JPHC-I	
11	Lee et al.	45	6	No‡	Experience-based		13	Self Defense Forces Health Study	
12	Shirota et al.	45	Open-ended	Yes	Experience-based		14		Filled in by an interviewer
13	Tokudome et al.	47	8	No‡	Data-based	15	16, 17	HERPACC, J-MICC	
14	Yamaoka et al.	65	7	Yes	Experience-based		18		Intakes of foods were queried for breakfast, lunch, and dinner, separately.

15	Wakai, Egami et al.	97	9	No‡	Data-based	19	19, 20	NISSIN project, LEMONADE	
16	Tokudome et al.	102	8	Yes	Data-based	21	22, 23	JADE Study	
17	Date et al.	122	Open-ended	Yes	Data-based	24	24		A “dish-based” (not “raw-food-based”) FFQ. Completed by an interviewer
18	Tsubono et al.	138	9	Yes	Data-based	25	11, 26–32	JPHC-I, II (5-year follow-up survey)	
19	Tsubono et al.	141	9	Yes	Data-based	33	33		
20	Sasaki et al.	150	12	Yes	Experience-based		34		
21	Shimizu et al.	169	8	Yes	Modification of an existing questionnaire		35	Takayama Study	Based on FFQ designed for the Multiethnic Cohort Study

*: Abbreviations: FFQ: food frequency questionnaire; HERPACC: Hospital-based Epidemiologic Research Program at Aichi Cancer Center; JACC: Japan Collaborative Cohort; JADE: Japanese Dietitians’ Epidemiologic; J-MICC: Japan Multi-Institutional Collaborative Cohort; JPHC: Japan Public Health Center-based Prospective Study; JPOS: Japanese Population-based Osteoporosis Study; LEMONADE: Longitudinal Evaluation of Multi-phasic, Odontological and Nutritional Associations in Dentists; NA: not available; NISSIN: New Integrated Suburban Seniority Investigation.

†: References for validity and reproducibility for main nutrients and/or food groups.

‡: Portion or serving sizes were asked for with selected foods.

The respondents to the questionnaire chose the intake frequency for each food item in 3 to 12 response categories. Open-ended questions for food frequency were used in 3 FFQs (Nos. 2, 12, and 17). Information on portion or serving sizes was collected in 12 (Nos. 2, 4–6, 12, 14, and 16–21) of the 21 FFQs. An additional 6 FFQs (Nos. 8–11, 13, and 15) included questions on the portion sizes for selected foods only. As shown in the “Main studies” column of Table 1, some of these FFQs have been used in cohort and cross-sectional studies in Japan.

The methods used to develop FFQs can be classified into 3 approaches. The first is the “experience-based” approach, in which experienced dietitians and/or epidemiologists select food items for the questionnaire (Nos. 1–6, 8–12, 14, and 20). In earlier FFQs (Nos. 2, 4, and 12), the intake frequencies and portion sizes of food groups were queried, instead of those of individual foods. In several cohort studies (Nos. 3 and 8–10), the FFQs were validated after a long follow-up of the cohorts, probably to utilize optimally the valuable cohort data by estimating the intakes of foods and/or nutrients using existing questionnaires.

The second method is the “data-based” approach (Nos. 13 and 15–19). Food items for FFQs are selected based on data from diet records so as to encompass defined percentages of the intakes of target nutrients.^1^^,^^19^^,^^21^^,^^24^^,^^25^^,^^33^ Additional criteria may be used to select food items, in order to fully explain inter-individual variations in the intakes of nutrients.^1^^,^^19^^,^^21^ This can be accomplished by using multiple regression analyses incorporating the consumption of individual food items as independent variables and the total intake of each nutrient as a dependent variable. In the regression analyses, food items are chosen by statistical variable selection, such as stepwise or forward methods. For their Takayama cohort study, Shimizu et al (FFQ No. 21) modified an existing FFQ, which had also been developed by using a data-based approach for a multiethnic cohort.^41^

The third method is the “short-version” approach (No. 7). In this method, a long FFQ is shortened by omitting food items. In this approach, the dietary intakes of target nutrients estimated by the long version are used instead of those derived from dietary records, as in the data-based approach. Food items for the short version are chosen from the food list of the long version, based on the between-person variations in nutrient intakes that can be explained by the items.

### Validity and reproducibility of FFQs in Japan

In the validation studies for FFQs (Table 2), participants kept dietary records for periods ranging from 1 to 63 days and completed the FFQs. Dietary intakes estimated with the questionnaires were compared with those derived from DRs. The median values of correlation coefficients (over target nutrients in each FFQ) between DRs and FFQs ranged from 0.31 to 0.56. The coefficient for fruit was always higher than that for vegetables, except for women in 1 FFQ (No. 18). To examine reproducibility, FFQs were completed twice in a period ranging from 3 days to 5 years, and estimated dietary intakes were then compared between the 2 FFQs. With intervals between 9 months to 1 year, the questionnaires were moderately reproducible: the median correlation coefficient for nutrients between the 2 FFQs ranged from 0.50 to 0.72. In a study in which the second questionnaire was administered 5 years after the first, the coefficient in men was considerably lower (median correlation coefficient for nutrients, 0.24). This may be due to actual changes in diet over such a long interval. In a pattern similar to that of nutrients, reproducibility was observed with vegetables and fruit.

**Table 2.** Summary of validation studies for food frequency questionnaires developed in Japan (sorted by number of food items included)*

No.	Authors of articles on validation studies	No. of food items	Participants	Correlation coefficients between DRs and FFQs (validity)								Correlation coefficients between 2 FFQs (reproducibility)			
	
				Duration of DR (days)	Number of nutrients	Adjustment for energy	Deattenuation	Nutrients (median [range])	Vegetables	Fruit		Nutrients (median [range])	Vegetables	Fruit	Interval between FFQs
1	Yatsuya et al.	9	47 men	6		Yes	Yes		0.11	0.38	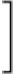		0.37	0.64	9 months
			47 women						0.36	0.38					
			47 female students						0.43	0.64					
2	Nakamura et al.	21	19 women	7	13	No	No	0.56 (0.27–0.90)	0.26	0.87					
3	Sauvaget et al.	22	1133 men	1		No	No			0.27					
			1872 women							0.20					
4	Katagiri et al.	24	36 men	7	11	No	No	0.31 (−0.09–0.46)				0.74 (0.61–0.91)			1 week
			36 women	7				0.46 (0.23–0.66)				0.63 (0.35–0.83)			
5	Sato et al.	26	74 women	1	1	No	No	0.51							
			(validity)		(calcium)										
			14 women									0.90			3 days
			(reproducibility)												
6	Uenishi et al.	28	208 women	3	10	No	No	0.44 (0.31–0.71)							
7	Takatsuka et al.	31	31 men and women	12	16	Yes	No	0.45 (−0.15–0.69)							
8	Ogawa et al.	40	55 men	12	15	Yes	Yes	0.43 (0.25–0.58)	0.60	0.76		0.49 (0.31–0.71)	0.43	0.50	1 year
			58 women					0.43 (0.30–0.69)	0.45	0.70		0.50 (0.40–0.64)	0.53	0.58	
9	Date et al.	40	85 men and women	12	34	Yes	No	0.31 (0.16–0.51)							
10	Tsubono et al.	44	94 men	28	30	Yes	No	0.36 (0.06–0.81)	0.27	0.55		0.24 (0.04–0.69)	0.37	0.40	5 years
			107 women					0.37 (0.11–0.52)	0.31	0.35		0.50 (0.27–0.60)	0.50	0.44	
11	Lee et al.	45	23 men	28	11	Yes	No	0.45 (0.19–0.63)	0.40	0.77					
12	Shirota et al.	45	65 men and women	7	15	No	No	0.52 (0.27–0.87)		0.40					
13	Tokudome, Imaeda	47	73 men	3	24	Yes	Yes	0.44 (0.12–0.86)							
	et al.		129 women		(validity)			0.38 (0.10–0.64)							
			(validity)												
			844 men		25							0.66 (0.55–0.74)		0.66	1 year
			1074 women		(reproducibility)							0.62 (0.54–0.73)		0.66	
			(reproducibility)												
14	Yamaoka et al.	65	71 men	7	18	Yes	No	0.35 (−0.10–0.65)	0.18†	0.82†		0.72 (0.54–0.81)	0.52†	0.64†	10 months
15	Wakai, Egami et al.	97	46 men	16	19	Yes	No	0.51 (0.12–0.73)	0.31	0.67	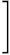	0.67 (0.48–0.82)	0.53	0.77	9 months
			42 women					0.51 (0.35–0.78)	0.52	0.66					
16	Tokudome, Imaeda	102	84 female dietitians	28	39	Yes	Yes	0.52 (0.28–0.73)		0.54					
	et al.				(validity)										
					36							0.55 (0.23–0.74)		0.61	1 year
					(reproducibility)										
17	Date et al.	122	67 men and women	56–63	13	Yes	No	0.46 (0.21–0.74)				0.72 (0.28–0.78)			1 week
18	Tsugane, Sasaki,	138	102 men‡	28	16	Yes	No	0.40 (0.22–0.82)	0.22	0.41		0.49 (0.30–0.82)	0.62	0.50	
	Ishihara et al.		113 women‡					0.39 (0.15–0.48)	0.32	0.23		0.50 (0.32–0.68)	0.53	0.50	
			174 men§	28	31	Yes	No	0.49 (0.26–0.65)	0.44	0.55		0.56 (0.46–0.77)	0.56	0.57	1 year
			176 women§					0.45 (0.18–0.64)	0.47	0.29		0.51 (0.33–0.72)	0.59	0.54	
19	Tsubono et al.	141	113 men and women	12	16	Yes	Yes	0.43 (0.24–0.85)				0.68 (0.47–0.91)			1 year
20	Sasaki et al.	150	47 women	3	18	Yes	Yes	0.48 (0.19–0.75)							
21	Shimizu et al.	169	58 men	3	14	Yes	No	0.43 (0.10–0.56)				0.62 (0.46–0.78)¶			1 year
			59 women					0.38 (0.10–0.66)				0.57 (0.13–0.67)¶			
			17 men	12	14	Yes	No‖	0.52 (0.18–0.86)							
			20 women					0.32 (0.03–0.77)							

*: Abbreviations: DR: diet record; FFQ: food frequency questionnaire; JPHC: Japan Public Health Center-based Prospective Study.

†: Energy from each food group.

‡: In JPHC-I area.

§: In JPHC-II area.

‖: The article reported de-attenuated correlation coefficients but did not include figures with both energy adjustment and de-attenuation. Energy-adjusted coefficients were therefore adopted.

¶: Intraclass correlation coefficient.

To determine the consumption of food groups and nutrients that are not easily estimated, the correlation coefficients for validity by food group (Table 3) and nutrient were summarized (Table 4). For food groups, the study by Sauvaget et al was excluded when medians of the coefficients over FFQs were computed, because that study used DRs with a duration of only 1 day as a standard, without accounting for within-person variations. For the same reason, the study by Sato and colleagues was also omitted in calculations of medians of the coefficients over FFQs for nutrients.

**Table 3.** Correlation coefficients between diet records and food frequency questionnaires by food group (validation studies were sorted by number of food items included)*

No.	Authors of articles on validation studies	Participants	Correlation coefficients between DRs and FFQs																

			Rice	Bread	Potatoes	Confectioneries	Fats and oils	Pulses	Fish and shellfish	Meat	Eggs	Milk or miilk plus dairy products	Vegetables	GYV	Other vegetables	Fruit	Mushrooms	Seaweed	Alcoholic beverages
1	Yatsuya et al.	47 men							0.24	0.37	0.50	0.62	0.11			0.38			0.44
		47 women				0.02			0.28	0.10	0.52	0.53	0.36			0.38			0.59
		47 female students				0.25			0.07	0.51	0.54	0.53	0.43			0.64			0.62
2	Nakamura et al.	19 women	0.81	0.81		0.78		0.39	0.73	0.47	0.71	0.93	0.26			0.87		0.72	
3	Sauvaget et al.	1133 men	0.29	0.32		0.15			0.17		0.19	0.29		0.15		0.27		0.18	
		1872 women	0.30	0.31		0.23			0.11		0.16	0.31		0.13		0.20		0.10	
8	Ogawa et al.	55 men				0.58		0.11		–0.10		0.71	0.60	0.54		0.76	0.32	0.44	0.70
		58 women				0.27		0.28		0.51		0.60	0.45	0.44		0.70	0.55	0.00	0.60
10	Tsubono et al.	94 men			0.24		0.17	0.39	0.37	0.18	0.25	0.46	0.27	0.25		0.55	0.30	0.21	0.75
		107 women			0.19		0.16	0.43	0.32	0.26	0.28	0.46	0.31	0.19		0.35	0.28	0.19	0.40
11	Lee et al.	23 men	0.56	0.80		0.40	0.30		0.51	0.48	0.69	0.58	0.40	0.40	0.35	0.77		0.56	0.91
12	Shirota et al.	65 men and women	0.92	0.73	0.02		0.16	0.15	0.58	0.32	0.39	0.53		0.58	0.33	0.40			
14	Yamaoka et al.	71 men				0.74	0.36	0.50	0.43	0.68	0.54	0.80	0.18			0.82			0.88
15	Wakai, Egami et al.	46 men	0.54	0.71	0.09	0.34	0.49	0.54	0.16	0.36	0.49	0.75	0.31	0.39	0.12	0.67	0.28	0.09	0.55
		42 women	0.65	0.35	0.09	0.37	0.57	0.66	0.33	0.61	0.42	0.69	0.52	0.57	0.42	0.66	0.48	0.18	0.62
16	Tokudome, Imaeda et al.	84 female dietitians	0.74			0.33	0.35	0.57	0.52	0.68	0.65	0.49		0.25	0.54	0.54		0.37	0.76
18	Tsugane, Sasaki,	102 men†			0.33	0.48	0.24	0.53	0.32	0.50	0.25	0.52	0.22	0.38		0.41	0.44	0.08	0.76
	Ishihara et al.	113 women†			0.20	0.38	0.21	0.49	0.32	0.45	0.42	0.64	0.32	0.32		0.23	0.38	0.06	0.50
		174 men‡			0.28	0.24	0.26	0.52	0.27	0.48	0.47	0.69	0.44	0.41		0.55	0.15	0.11	0.05
		176 women‡			0.30	0.26	0.28	0.54	0.23	0.44	0.45	0.64	0.47	0.37		0.29	0.12	0.18	0.49

Median§			0.74	0.77	0.15	0.38	0.30	0.46	0.43	0.47	0.52	0.61	0.37	0.40	0.34	0.60	0.34	0.22	0.62

*: Abbreviations: DR: diet record; FFQ: food frequency questionnaire; GYV: green-yellow vegetables; JPHC: Japan Public Health Center-based Prospective Study.

†: In JPHC-I area.

‡: In JPHC-II area.

§: Medians over FFQs. When 2 or more values were available, ie, for sex and/or population, for 1 FFQ, their median was adopted as the representative value for that FFQ. The study by Sauvaget et al. was excluded since it used DRs conducted for only 1 day as a standard and did not account for intra-individual variations.

**Table 4.** Correlation coefficients between diet records and food frequency questionnaires by nutrient (validation studies were sorted by number of food items included)*

No.	Authors of articles on validation studies	Participants	Correlation coefficients between DRs and FFQs											

			Energy	Protein	Fat	Carbo- hydrate	Calcium	Iron	Potassium	Phosphorus	Sodium or NaCl	Vitamin A	Retinol	Carotene
2	Nakamura et al.	19 women	0.43	0.44	0.34	0.76	0.90	0.49			0.31	0.65	0.83	0.56
4	Katagiri et al.	36 men	0.38	0.38	0.11	0.40	0.46	−0.09			0.13	0.44		
		36 women	0.57	0.37	0.57	0.64	0.35	0.39			0.23	0.46		
5	Sato et al.	74 women					0.51							
6	Uenishi et al.	208 women	0.42	0.32	0.31	0.39	0.68	0.50			0.47	0.71		
7	Takatsuka et al.	31 men and women	0.55	0.57	−0.03	0.34	0.69				0.33	0.22	0.21	0.45
8	Ogawa et al.	55 men	0.55	0.25	0.37	0.57	0.57	0.35	0.45	0.52	0.37		0.38	0.56
		58 women	0.36	0.49	0.50	0.43	0.67	0.47	0.45	0.69	0.33		0.30	0.45
9	Date et al.	85 men and women	0.20	0.24	0.46		0.35	0.28	0.38		0.31	0.35		
10	Tsubono et al.	94 men	0.52	0.28	0.30	0.51	0.56	0.31	0.38	0.56	0.33		0.36	
		107 women	0.38	0.34	0.41	0.33	0.37	0.30	0.37	0.44	0.49		0.34	
11	Lee et al.	23 men	0.23	0.44	0.19	0.45	0.52	0.31	0.63			0.19		
12	Shirota et al.	65 men and women	0.87	0.71	0.52	0.87	0.42	0.46	0.53		0.32	0.28		
13	Tokudome, Imaeda	73 men	0.49	0.50	0.62	0.86	0.49	0.58				0.27		0.39
	et al.	129 women	0.44	0.36	0.48	0.64	0.59	0.44				0.22		0.38
14	Yamaoka et al.	71 men	0.64	0.16	0.65	0.56	0.55	0.14	−0.10	0.26	0.34	0.36		
15	Wakai, Egami et al.	46 men	0.21	0.24	0.60	0.46	0.71	0.12	0.57			0.49	0.56	0.33
		42 women	0.38	0.53	0.50	0.53	0.78	0.52	0.73			0.45	0.36	0.46
16	Tokudome, Imaeda et al.	84 female dietitians	0.48	0.53	0.49	0.57	0.64	0.55	0.61	0.58		0.35		0.33
17	Date et al.	67 men and women	0.65			0.58	0.74		0.50		0.26	0.21	0.53	0.25
18	Tsugane, Sasaki,	102 men†	0.55	0.30	0.52	0.56	0.43	0.49	0.39	0.37	0.41		0.22	0.36
	Ishihara et al.	113 women†	0.44	0.27	0.46	0.37	0.47	0.33	0.31	0.42	0.48		0.43	0.33
		174 men‡	0.34	0.30	0.57	0.59	0.65	0.54	0.49	0.49	0.42		0.35	0.47
		176 women‡	0.22	0.31	0.40	0.39	0.64	0.51	0.49	0.54	0.45		0.47	0.49
19	Tsubono et al.	113 men and women	0.49	0.29	0.50	0.55	0.60	0.30	0.43	0.47	0.33		0.36	0.38
20	Sasaki et al.	47 women	0.48	0.48	0.55	0.48	0.49	0.40	0.68	0.59	0.32	0.38		
21	Shimizu et al.	58 men	0.38	0.45	0.43	0.51	0.51				0.18	0.42		0.36
		59 women	0.25	0.37	0.51	0.29	0.59				0.10	0.27		0.48
		17 men	0.44	0.78	0.26	0.38	0.86				0.28	0.47		0.58
		20 women	0.49	0.67	0.14	0.24	0.77				0.22	0.19		0.28

Median§			0.46	0.39	0.46	0.50	0.58	0.40	0.48	0.50	0.33	0.35	0.38	0.41

No.	Authors of articles on validation studies	Participants	Correlation coefficients between DRs and FFQs												

			Vitamin B_1_	Vitamin B_2_	Niacin	Vitamin C	Vitamin D	Vitamin E	SFA	MUFA	PUFA	n-6 PUFA	n-3 PUFA	Cholesterol	Dietary fiber

2	Nakamura et al.	19 women	0.27	0.76		0.77									
4	Katagiri et al.	36 men	0.15	0.31		0.23									
		36 women	0.66	0.39		0.49									
6	Uenishi et al.	208 women					0.40								
7	Takatsuka et al.	31 men and women				0.44	0.53	0.50	0.51	0.12	−0.15			0.52	
8	Ogawa et al.	55 men	0.33	0.43	0.33	0.58									
		58 women	0.31	0.54	0.47	0.43									
9	Date et al.	85 men and women	0.36	0.31		0.27			0.50	0.36	0.15	0.16	0.21	0.29	
10	Tsubono et al.	94 men	0.36	0.43	0.14	0.38			0.42	0.23	0.06	0.14	0.20	0.36	
		107 women	0.22	0.39	0.11	0.29			0.50	0.39	0.22	0.15	0.35	0.30	
11	Lee et al.	23 men				0.35									0.56
12	Shirota et al.	65 men and women	0.74	0.58		0.35									
13	Tokudome, Imaeda	73 men	0.26	0.57		0.45	0.65	0.31	0.64	0.43	0.44	0.12	0.55	0.13	0.36
	et al.	129 women	0.10	0.43		0.52	0.40	0.17	0.42	0.34	0.25	0.31	0.23	0.19	0.47
14	Yamaoka et al.	71 men	0.42	0.37	−0.07	0.52	0.37	0.44						0.19	
15	Wakai, Egami et al.	46 men				0.55		0.58	0.73	0.63	0.39			0.50	0.51
		42 women				0.53		0.41	0.48	0.53	0.49			0.35	0.64
16	Tokudome, Imaeda et al.	84 female dietitians				0.42	0.64	0.32	0.62	0.44	0.28	0.32	0.29	0.59	0.65
17	Date et al.	67 men and women				0.38		0.42							
18	Tsugane, Sasaki,	102 men†	0.40	0.34	0.35	0.42			0.61	0.50	0.27	0.30	0.21	0.33	0.45
	Ishihara et al.	113 women†	0.41	0.45	0.15	0.22			0.60	0.44	0.24	0.21	0.34	0.35	0.44
		174 men‡	0.28	0.55	0.33	0.46			0.62	0.55	0.44	0.49	0.30	0.47	0.57
		176 women‡	0.32	0.55	0.22	0.44			0.51	0.37	0.33	0.42	0.19	0.47	0.49
19	Tsubono et al.	113 men and women	0.24	0.52	0.37	0.42									
20	Sasaki et al.	47 women	0.46	0.58	0.19	0.45			0.75	0.50	0.37			0.49	
21	Shimizu et al.	58 men				0.21		0.29						0.36	
		59 women				0.21		0.39						0.31	
		17 men				0.55		0.71						0.18	
		20 women				0.46		0.34						0.49	

Median§			0.36	0.50	0.23	0.42	0.53	0.42	0.57	0.41	0.29	0.22	0.28	0.37	0.56

*: Abbreviations: DR: diet record; FFQ: food frequency questionnaire; SFA: saturated fatty acids; MUFA: monounsaturated fatty acids; PUFA: polyunsaturated fatty acids; JPHC: Japan Public Health Center-based Prospective Study.

†: In JPHC-I area.

‡: In JPHC-II area.

§: Medians over FFQs. When 2 or more values were available (for sex and/or population) for 1 FFQ, their median was adopted as the representative value of that FFQ. The study by Sato et al. was excluded since it used DRs conducted for only 1 day as a standard and did not account for intra-individual variations.

The validity for reported consumption of a food group was high (median of correlation coefficients for all studies, ≥0.60) for rice, bread, milk or milk plus dairy products, fruit, and alcoholic beverages; moderate (0.40–0.59) for pulses, fish and shellfish, meat, eggs, and green-yellow vegetables; and fair (0.30–0.39) for confectioneries, fats and oils, total vegetables, vegetables other than green-yellow vegetables, and mushrooms (Table 3). It was, however, poor (median of correlation coefficients for all FFQs, <0.30) for potatoes and seaweed. For most nutrients, including energy, the median of correlation coefficients over studies for validity was distributed from 0.40 to 0.59 (Table 4). The validity was fair (median of correlation coefficients over studies, 0.30–0.39) for protein, sodium or NaCl, retinol, cholesterol, and vitamins A and B_1_; and poor (<0.30) for niacin and polyunsaturated fatty acids (PUFA). Among fatty acids, the validity was highest for saturated fatty acids (SFA), followed by monounsaturated fatty acids (MUFA) and PUFA.

### Short FFQs versus Long FFQs

When FFQs that were developed and validated in Japan are sorted by the number of included food items (Table 1), they can be divided into long FFQs, with 97 or more food items (Nos. 15–21), and short FFQs, with fewer than 70 items (Nos. 1–14). The FFQs in the former group were principally developed using a data-based approach, whereas most FFQs in the latter group were devised based on the experience of dietitians and/or epidemiologists. A dotted line is inserted in Table 1 to show these 2 groups.

Did a longer, more systematically prepared FFQ result in higher validity? To address this issue, the validity of long and short FFQs was compared by examining the medians of correlation coefficients between DRs and FFQ estimates for nutrients. To ensure comparability, the analysis was limited to FFQs for which energy-adjusted correlation coefficients had been reported, and either DRs of 7 or more days had been collected or the de-attenuation of within-person variation of nutrient intakes^42^ had been conducted. A very short-duration DR with no de-attenuation would have resulted in apparently lower validity for the assessment of usual or average diets investigated over a long period among participants. Energy-adjusted coefficients were used for the analysis because dietary intakes estimated by FFQs are often adjusted for total energy intake in nutritional epidemiology,^43^ in order to account for the confounding of energy intake and to adjust for general over-reporting or under-reporting of food intake in FFQs. In addition, FFQs designed to estimate nutrients relevant only to a disease (osteoporosis, Nos. 5 and 6) were also excluded.

**Figure 1.**
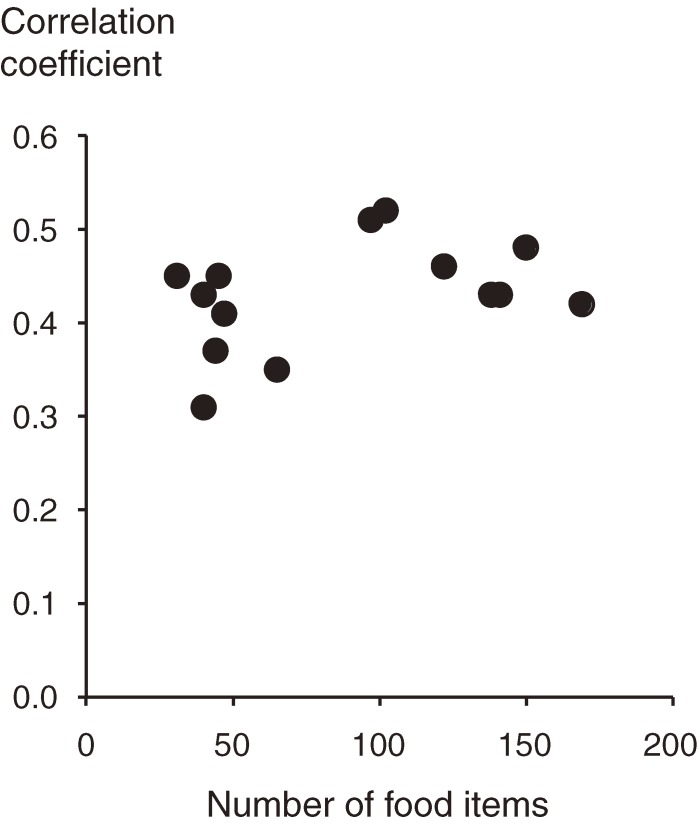
Association between the number of food items and the correlation coefficients between diet records and food frequency questionnaires (FFQs). The analysis was limited to FFQs for which energy-adjusted correlation coefficients had been reported, and either DRs of 7 or more days had been collected or the de-attenuation of within-person variation of nutrient intakes had been conducted. FFQs designed to estimate nutrients relevant only to a disease were excluded

In long FFQs, the correlation coefficients were slightly higher and encompassed a narrower range than those of short FFQs. In long FFQs, the median correlation coefficient for nutrients in an individual FFQ ranged from 0.42 to 0.52 (median of the medians, 0.46) (Nos. 15–21); it ranged from 0.31 to 0.45 (median, 0.41) in short FFQs (Nos. 7–11, 13, and 14). This result can be clearly seen in Figure 1, which shows the association between number of food items and correlation coefficient. When FFQs without energy-adjusted correlation coefficients (Nos. 2, 4, and 12) were also included in the analysis, the medians of correlation coefficients were more widely distributed (range, 0.31–0.56), although the median of the medians remained nearly identical (0.42).

## DISCUSSION

In the present review, more than 20 FFQs developed and validated in Japan were identified. They were reasonably valid and reproducible, though relatively poor validity was observed in FFQ estimates for several food groups and nutrients. The questionnaires could be divided into long and short FFQs, and the former had slightly higher validity for estimates of nutrient intake.

A limitation of this review was that the FFQs and studies of their validity differed in characteristics such as the target nutrients and food groups, the population investigated in the validation studies, the period encompassed by the DR, and the statistical analyses of validation data (eg, energy adjustment, de-attenuation). It is not possible to accurately summarize the correlation coefficients for validity and reproducibility abstracted from published articles because the abovementioned factors may have affected the measures. This review, therefore, should be considered a rough description of the validity and reproducibility of the identified FFQs, which were analyzed in their entirety, and by food group, nutrient, and FFQ length.

The median of correlation coefficients between DRs and FFQ varied between 0.42 and 0.52 for energy-adjusted nutrients, even for long, comprehensive FFQs; this is substantially lower than corresponding figures from Western countries, which range from 0.6 to 0.7.^44^ This suggests that it may be more difficult to accurately assess complicated modern Japanese diets, which can include traditional Japanese, Western, and Chinese foods and dishes.

Considerable variation was observed in the correlation coefficients between DRs and FFQs among food groups. This may in part be due to differences in the number of food items, ability to recall intake frequencies and portion sizes, the wording of questions in the FFQ, and between-person variations in consumption among food groups. To take one example, the validity was higher for fruits than for vegetables, which may be due to the inclusion of fewer items in the fruit group. In addition, it might be easier for respondents to report intake frequencies and portion sizes of fruits than those of vegetables since fruits are more often consumed as raw foods instead of cooked dishes. Most FFQs included more detailed questions on rice, as compared with other food items, probably to more accurately assess the consumption of this main staple in Japan, which would result in higher validity. If the between-person variation in a food group intake is large, the correlation coefficient for validity will be increased. This may be true, say, for alcoholic beverages: some individuals drink no alcohol, while others consume it heavily. More detailed questionnaires, with more items in a food group, may be needed to accurately evaluate the consumption of food groups for which the validity tends to be low.

When comparing the health effects among food groups based on findings from studies using FFQs, one should take into account differences in the validity of FFQs. Vegetables and fruit are often contrasted in terms of their associations with cancer risk.^45^ The higher validity for fruit than for vegetables, however, may lead to a seemingly stronger association between cancer incidence and fruit intake.

With regard to nutrients, the validity for sodium or NaCl, niacin, and PUFA was comparatively low. Using FFQs to measure the dietary intake of sodium and PUFA is not straightforward, as it requires assessing the use of seasonings and cooking oils, respectively. Seasonings are major contributors to sodium intake, as cooking oils are to PUFA intake.^46^ Indeed, the correlation coefficient between DRs and FFQ was rather low for fats and oils (median over FFQs, 0.30). Although some FFQs include detailed questions on the use of seasonings and cooking oils,^25^^,^^34^ there is scarce evidence indicating that FFQs are improved by the addition of these supplementary questions. Measuring fatty acids in phospholipid fractions of plasma or in erythrocyte membranes may be required to validly assess long-term average PUFA intake.^47^ It is not clear why the validity for niacin was low. According to Tsubono et al,^25^ rice is a major contributor to niacin intake, followed by pork, chicken, and tuna. Since the correlation between DR and FFQ estimates for rice was high, there may be considerable measurement errors in the other foods.

For nutrients, the validity of long FFQs was somewhat higher than that of short FFQs; the median of correlation coefficients between DRs and FFQ estimates was higher in the former group by 0.05 (0.46 vs 0.41). In nutritional epidemiology, to estimate relative risk, participants with higher intakes of a nutrient are frequently compared to those with lower intakes. The random measurement error of FFQ will reduce the relative risk. If the true intake of a nutrient and that estimated by an FFQ have the same standard deviation, the observed relative risk (RR_o_) can be computed as follows^42^:

$${\mathrm{RR}}_{o}={\left({\mathrm{RR}}_{t}\right)}^{\gamma }$$

where RR_t_ is the true relative risk, and *γ* is the correlation coefficient between the true intake and the one derived from an FFQ. When *γ* is assumed to be 0.46 and 0.41 for the long and short FFQs, respectively (ie, the median of correlation coefficients over FFQs for nutrients), and the RR_t_ to be 2, the corresponding values for RR_o_ would be 1.38 and 1.33. Even if RR_t_ is assumed to be 3, the respective values for RR_o_ would be 1.66 and 1.57. Any additional gain in information that might be obtained by using more detailed questionnaires appears to be small if these assumptions are correct.

Of course, long, comprehensive FFQs have more value than simply increasing validity. They enable researchers to assess the intakes of a greater number of individual foods, food groups, and nutrients. Since foods contributing to nutrient intake vary depending on the target nutrients they possess, the food list in the questionnaire must be extended to estimate the intakes of more nutrients. Furthermore, the data-based approach that is often used to develop comprehensive FFQs may lead to more stable validity, as suggested by the narrower range of validity in long FFQs, as compared with short FFQs. Long FFQs, however, impose a heavier burden on study participants because they require more time to complete. The cost/benefit ratio must therefore be considered in light of the study aims.

In summary, FFQs are a useful tool to assess dietary intakes in Japan. However, their validity tends to be low for several food groups and nutrients. Careful consideration must be given to the measurement of such dietary variables and to the interpretation of data.

## References

[1] Willett W. Food-frequency methods. In: Willett W, editor. Nutritional epidemiology. 2nd ed. New York, Oxford: Oxford University Press; 1998. p. 74–100.

[2] Yatsuya H, Ohwaki A, Tamakoshi K, Wakai K, Koide K, Otsuka R, et al. Reproducibility and validity of a simple checklist-type questionnaire for food intake and dietary behavior. J Epidemiol 2003;13:235–45. 14604218 10.2188/jea.13.235 PMC9691396

[3] Nakamura M, Aoki N, Nasu K, Kondo I A comparison between a food frequency and amount questionnaire and 7-day diet record with weighing. Jpn J Public Health 1994;41:682–92 (in Japanese). 7949280

[4] Sauvaget C, Allen N, Hayashi M, Spencer E, Nagano J. Validation of a food frequency questionnaire in the Hiroshima/Nagasaki Life Span Study. J Epidemiol 2002;12:394–401. 12395883 10.2188/jea.12.394 PMC10635806

[5] Katagiri A, Hashimoto S, Ohashi Y, Shirogane K, Sakamoto N, Makimoto S Reproducibility and validity of a semi-quantitative food frequency questionnaire. Jpn J Public Health 1998;45:1127–36 (in Japanese). 10067079

[6] Sato Y, Tamaki J, Kitayama F, Kusaka Y, Kodera Y, Koutani A, et al. Development of a food-frequency questionnaire to measure the dietary calcium intake of adult Japanese women. Tohoku J Exp Med 2005;207:217–22. 10.1620/tjem.207.217 16210833

[7] Uenishi K, Ishida H, Nakamura K. Development of a simple food frequency questionnaire to estimate intakes of calcium and other nutrients for the prevention and management of osteoporosis. J Nutr Sci Vitaminol (Tokyo) 2008;54:25–9. 10.3177/jnsv.54.25 18388404

[8] Takatsuka N, Kurisu Y, Nagata C, Owaki A, Kawakami N, Shimizu H. Validation of simplified diet history questionnaire. J Epidemiol 1997;7:33–41. 9127571 10.2188/jea.7.33

[9] Ogawa K, Tsubono Y, Nishino Y, Watanabe Y, Ohkubo T, Watanabe T, et al. Validation of a food-frequency questionnaire for cohort studies in rural Japan. Public Health Nutr 2003;6:147–57. 10.1079/PHN2002411 12675957

[10] Date C, Fukui M, Yamamoto A, Wakai K, Ozeki A, Motohashi Y, et al. Reproducibility and validity of a self-administered food frequency questionnaire used in the JACC study. J Epidemiol 2005;15 Suppl 1:S9–23. 10.2188/jea.15.S9 15881192 PMC8565864

[11] Tsugane S, Sasaki S, Kobayashi M, Tsubono Y, Akabane M. Validity and reproducibility of the self-administered food frequency questionnaire in the JPHC Study Cohort I: study design, conduct and participant profiles. J Epidemiol 2003;13 Suppl 1:S2–12. 12701628 10.2188/jea.13.1sup_2 PMC9767693

[12] Tsubono Y, Kobayashi M, Sasaki S, Tsugane S. Validity and reproducibility of a self-administered food frequency questionnaire used in the baseline survey of the JPHC Study Cohort I. J Epidemiol 2003;13 Suppl 1:S125–33. 12701640 10.2188/jea.13.1sup_125 PMC9767689

[13] Lee KY, Uchida K, Shirota T, Kono S. Validity of a self-administered food frequency questionnaire against 7-day dietary records in four seasons. J Nutr Sci Vitaminol (Tokyo) 2002;48:467–76. 12775113 10.3177/jnsv.48.467

[14] Shirota T, Yoshizumi F A study on convenient dietary assessment. Jpn J Public Health 1990;37:100–8 (in Japanese). 2131966

[15] Tokudome S, Goto C, Imaeda N, Tokudome Y, Ikeda M, Maki S. Development of a data-based short food frequency questionnaire for assessing nutrient intake by middle-aged Japanese. Asian Pac J Cancer Prev 2004;5:40–3. 15075003

[16] Tokudome Y, Goto C, Imaeda N, Hasegawa T, Kato R, Hirose K, et al. Relative validity of a short food frequency questionnaire for assessing nutrient intake versus three-day weighed diet records in middle-aged Japanese. J Epidemiol 2005;15:135–45. 10.2188/jea.15.135 16141632 PMC7851066

[17] Imaeda N, Goto C, Tokudome Y, Hirose K, Tajima K, Tokudome S. Reproducibility of a short food frequency questionnaire for Japanese general population. J Epidemiol 2007;17:100–7. 10.2188/jea.17.100 17545697 PMC7058456

[18] Yamaoka K, Tango T, Watanabe M, Yokotsuka M Validity and reproducibility of a semi-quantitative food frequency questionnaire for nutritional education of patients of diabetes mellitus (FFQW65). Jpn J Public Health 2000;47:230–44 (in Japanese). 10783633

[19] Wakai K, Egami I, Kato K, Lin Y, Kawamura T, Tamakoshi A, et al. A simple food frequency questionnaire for Japanese diet—Part I. Development of the questionnaire, and reproducibility and validity for food groups. J Epidemiol 1999;9:216–26. 10510578 10.2188/jea.9.216

[20] Egami I, Wakai K, Kato K, Lin Y, Kawamura T, Tamakoshi A, et al. A simple food frequency questionnaire for Japanese diet—Part II. Reproducibility and validity for nutrient intakes. J Epidemiol 1999;9:227–34. 10510579 10.2188/jea.9.227

[21] Tokudome S, Ikeda M, Tokudome Y, Imaeda N, Kitagawa I, Fujiwara N. Development of data-based semi-quantitative food frequency questionnaire for dietary studies in middle-aged Japanese. Jpn J Clin Oncol 1998;28:679–87. 10.1093/jjco/28.11.679 9861235

[22] Tokudome S, Imaeda N, Tokudome Y, Fujiwara N, Nagaya T, Sato J, et al. Relative validity of a semi-quantitative food frequency questionnaire versus 28 day weighed diet records in Japanese female dietitians. Eur J Clin Nutr 2001;55:735–42. 10.1038/sj.ejcn.1601215 11528486

[23] Imaeda N, Fujiwara N, Tokudome Y, Ikeda M, Kuriki K, Nagaya T, et al. Reproducibility of a semi-quantitative food frequency questionnaire in Japanese female dietitians. J Epidemiol 2002;12:45–53. 11848184 10.2188/jea.12.45 PMC10432251

[24] Date C, Yamaguchi M, Tanaka H. Development of a food frequency questionnaire in Japan. J Epidemiol 1996;6 Suppl 3:S131–6. 8800285 10.2188/jea.6.3sup_131

[25] Tsubono Y, Takamori S, Kobayashi M, Takahashi T, Iwase Y, Iitoi Y, et al. A data-based approach for designing a semiquantitative food frequency questionnaire for a population-based prospective study in Japan. J Epidemiol 1996;6:45–53. 8795957 10.2188/jea.6.45

[26] Sasaki S, Kobayashi M, Ishihara J, Tsugane S. Self-administered food frequency questionnaire used in the 5-year follow-up survey of the JPHC Study: questionnaire structure, computation algorithms, and area-based mean intake. J Epidemiol 2003;13 Suppl 1:S13–22. 12701629 10.2188/jea.13.1sup_13 PMC9767697

[27] Tsugane S, Kobayashi M, Sasaki S. Validity of the self-administered food frequency questionnaire used in the 5-year follow-up survey of the JPHC Study Cohort I: comparison with dietary records for main nutrients. J Epidemiol 2003;13 Suppl 1:S51–6. 12701631 10.2188/jea.13.1sup_51 PMC9767699

[28] Sasaki S, Kobayashi M, Tsugane S. Validity of a self-administered food frequency questionnaire used in the 5-year follow-up survey of the JPHC Study Cohort I: comparison with dietary records for food groups. J Epidemiol 2003;13 Suppl 1:S57–63. 12701632 10.2188/jea.13.1sup_57 PMC9767694

[29] Kobayashi M, Sasaki S, Kawabata T, Hasegawa K, Tsugane S. Validity of a self-administered food frequency questionnaire used in the 5-year follow-up survey of the JPHC Study Cohort I to assess fatty acid intake: comparison with dietary records and serum phospholipid level. J Epidemiol 2003;13 Suppl 1:S64–81. 12701633 10.2188/jea.13.1sup_64 PMC9767695

[30] Sasaki S, Matsumura Y, Ishihara J, Tsugane S. Validity of a self-administered food frequency questionnaire used in the 5-year follow-up survey of the JPHC Study Cohort I to assess dietary fiber intake: comparison with dietary records. J Epidemiol 2003;13 Suppl 1:S106–14. 12701638 10.2188/jea.13.1sup_106 PMC9767692

[31] Sasaki S, Ishihara J, Tsugane S. Reproducibility of a self-administered food frequency questionnaire used in the 5-year follow-up survey of the JPHC Study Cohort I to assess food and nutrient intake. J Epidemiol 2003;13 Suppl 1:S115–24. 12701639 10.2188/jea.13.1sup_115 PMC9767696

[32] Ishihara J, Sobue T, Yamamoto S, Yoshimi I, Sasaki S, Kobayashi M, et al. Validity and reproducibility of a self-administered food frequency questionnaire in the JPHC Study Cohort II: study design, participant profile and results in comparison with Cohort I. J Epidemiol 2003;13 Suppl 1:S134–47. 12701641 10.2188/jea.13.1sup_134 PMC9767691

[33] Tsubono Y, Ogawa K, Watanabe Y, Nishino Y, Tsuji I, Watanabe T, et al. Food frequency questionnaire as a screening test. Nutr Cancer 2001;39:78–84. 10.1207/S15327914nc391_11 11588906

[34] Sasaki S, Yanagibori R, Amano K. Self-administered diet history questionnaire developed for health education: a relative validation of the test-version by comparison with 3-day diet record in women. J Epidemiol 1998;8:203–15. 9816812 10.2188/jea.8.203

[35] Shimizu H, Ohwaki A, Kurisu Y, Takatsuka N, Ido M, Kawakami N, et al. Validity and reproducibility of a quantitative food frequency questionnaire for a cohort study in Japan. Jpn J Clin Oncol 1999;29:38–44. 10.1093/jjco/29.1.38 10073150

[36] Wakai K, Kawamura T, Matsuo S, Hotta N, Ohno Y. Risk factors for IgA nephropathy: a case-control study in Japan. Am J Kidney Dis 1999;33:738–45. 10.1016/S0272-6386(99)70228-3 10196018

[37] Wakai K, Takashi M, Okamura K, Yuba H, Suzuki K, Murase T, et al. Foods and nutrients in relation to bladder cancer risk: a case-control study in Aichi Prefecture, Central Japan. Nutr Cancer 2000;38:13–22. 10.1207/S15327914NC381_3 11341038

[38] Wakai K, Okamoto K, Tamakoshi A, Lin Y, Nakayama T, Ohno Y. Seasonal allergic rhinoconjunctivitis and fatty acid intake: a cross-sectional study in Japan. Ann Epidemiol 2001;11:59–64. 10.1016/S1047-2797(00)00182-4 11164121

[39] Wakai K, Nakai S, Matsuo S, Kawamura T, Hotta N, Maeda K, et al. Risk factors for IgA nephropathy: a case-control study with incident cases in Japan. Nephron 2002;90:16–23. 10.1159/000046309 11744800

[40] Sakamoto N, Kono S, Wakai K, Fukuda Y, Satomi M, Shimoyama T, et al. Dietary risk factors for inflammatory bowel disease: a multicenter case-control study in Japan. Inflamm Bowel Dis 2005;11:154–63. 10.1097/00054725-200502000-00009 15677909

[41] Stram DO, Hankin JH, Wilkens LR, Pike MC, Monroe KR, Park S, et al. Calibration of the dietary questionnaire for a multiethnic cohort in Hawaii and Los Angeles. Am J Epidemiol 2000;151:358–70. 10695594 10.1093/oxfordjournals.aje.a010214 PMC4482461

[42] Willett W. Correction for the effects of measurement error. In: Willett W, editor. Nutritional epidemiology. 2nd ed. New York, Oxford: Oxford University Press; 1998. p. 302–20.

[43] Willett W, Stampfer M. Implications of total energy intake for epidemiologic analyses. In: Willett W, editor. Nutritional epidemiology. 2nd ed. New York, Oxford: Oxford University Press; 1998. p. 273–301.

[44] Willett W, Lenart E. Reproducibility and validity of food-frequency questionnaires. In: Willett W, editor. Nutritional epidemiology. 2nd ed. New York, Oxford: Oxford University Press; 1998. p. 101–47.

[45] International Agency for Research on Cancer, World Health Organization. IARC Handbooks of Cancer Prevention, vol. 8: Fruit and Vegetables. Lyon: IARC Press; 2003.

[46] Tokudome Y, Imaeda N, Ikeda M, Kitagawa I, Fujiwara N, Tokudome S. Foods contributing to absolute intake and variance in intake of fat, fatty acids and cholesterol in middle-aged Japanese. J Epidemiol 1999;9:78–90. 10337080 10.2188/jea.9.78

[47] Hunter D. Biochemical indicators of dietary intake. In: Willett W, editor. Nutritional epidemiology. 2nd ed. New York, Oxford: Oxford University Press; 1998. p. 174–243.

